# A minireview of the medicinal and edible insects from the traditional Chinese medicine (TCM)

**DOI:** 10.3389/fphar.2023.1125600

**Published:** 2023-03-16

**Authors:** Enming Zhang, Xin Ji, Fang Ouyang, Yang Lei, Shun Deng, Haibo Rong, Xuangen Deng, Hai Shen

**Affiliations:** ^1^ School of Sports Medicine and Physical Therapy, Beijing Sport University, Beijing, China; ^2^ Institute of Zoology, Chinese Academy of Science, Beijing, China; ^3^ College of Arts and Sciences, Boston University, Boston, MA, United States; ^4^ Sichuan Provincial Orthopedic Hospital, Chengdu, China

**Keywords:** entomoceuticals, folk medicine, medicinal insects, health food, insect omics

## Abstract

Entomoceuticals define a subset of pharmaceuticals derived from insects. The therapeutic effect of insect-derived drugs has been empirically validated by the direct use of various folk medicines originating from three sources in particular: the glandular secretions of insects (e.g., silk, honey, venom), the body parts of the insect or the whole used live or by various processing (e.g., cooked, toasted, ground), and active ingredients extracted from insects or insect-microbe symbiosis. Insects have been widely exploited in traditional Chinese medicine (TCM) relative to other ethnomedicines, especially in the prospect of insect species for medicinal uses. It is noticeable that most of these entomoceuticals are also exploited as health food for improving immune function. In addition, some edible insects are rich in animal protein and have high nutritional value, which are used in the food field, such as insect wine, health supplements and so on. In this review, we focused on 12 insect species that have been widely used in traditional Chinese herbal formulae but have remained less investigated for their biological properties in previous studies. We also combined the entomoceutical knowledge with recent advances in insect omics. This review specifies the underexplored medicinal insects from ethnomedicine and shows their specific medicinal and nutritional roles in traditional medicine.

## Introduction

Insecta comprises a myriad of insect species, far exceeding the total number of plants and other animals, and is estimated at approximately 5.5 million (Stork, 2018). Insects have adapted to multiple survival strategies against extreme environmental conditions, plant-derived repellents, hazardous pathogens, and natural predators (Schmid-Hempel, 2005). For a long time, insects have been widely exploited by mankind for food, clothing, ornament, and medicine (Costa-Neto and Dunkel, 2016; Singh et al., 2016; Mishra, 2017). The substances isolated from insects, such as silk, honey, venom, and even the processed whole body or specific organ of the insect, have been promised as potential drug candidates based on empirical therapies. Some countries, such as China, Korea, India, Mexico, and Brazil, have a long history of insect-derived crude drugs in their folk medicines (Tsuneo et al., 1988; Pemberton, 1999; Costa-Neto, 2002). However, in recent decades, insects have been less prospected by modern analytic techniques than plants, microbes, mollusks, and even arachnids (Harvey, 2000). It was estimated that insects have been consumed in different ways by about 2.5 billion people, predominantly in parts of Asia, Africa and Latin America, with over 2,100 species cataloged as edible (Jantzen da Silva Lucas et al., 2020). It was reported that insects have been consumed in 11 European countries, 14 countries in Oceania, 23 American countries, 29 Asian countries, and 35 African countries. Mexico, China, Thailand, and India are the leading consumption countries and the most edible insect species are from the orders of Hymenoptera and Coleoptera (Melgar-Lalanne et al., 2019). Today, insects have become an alternative food with high nutritional value and few negative environmental impacts that were further recommended by the Food and Agriculture Organization (FAO) in 2013 (Huis et al., 2013; Ball, 2014). Similar to the fatty acids in poultry and fish, insects such as bees, ants, butterflies, moths, beetles, grasshoppers and locusts are between 7 and 77 g/100 g dry weight and the caloric value of those insects varies between 293 and 762 kcal/100 g (Verkerk et al., 2007). In addition, the mineral content of those insects ranges from 3 to 8 g/100 g and the calcium concentration is around 920 mg/100 g dry weight, and they also contain a much higher amount of zinc and iron than beef. In conclusion, insects contain high nutrient content including protein, fat, amino acids, fatty acids, minerals, and vitamins (Verkerk et al., 2007). In recent years, an increasing number of studies have searched for novel bioactive molecules from insects for medical purposes both in China and other countries.

Archaic knowledge of the medicinal use of insects is unexpectedly abundant in most regions with well-preserved folklore medicine. The ethnoentomological culture was well described across Latin America, Mexico, Africa, India, China and South Korea, involving entomophagy, entomotherapy and the use of insects in rite-of-passage rituals. Costa-Neto reported that in the northeast Brazilian state of Bahia, at least 42 species belonging to nine insect orders were used as folk medicines (Costa-Neto, 2002), and a survey solicited from the Mexican folk medicine noted 43 species of insects assorting into six orders (Pino Moreno and Pino Moreno, 1988). In two tribal societies of northeast India, it was demonstrated that 12 insect species are deemed therapeutically valuable and are used by local tribes to treat a variety of disorders in humans and domestic animals (Chakravorty et al., 2011). Approximately 300 insect species in relation to 13 orders were officially written into more than 1,700 classical Chinese herbal prescriptions, which are still being used by modern Chinese people (Feng et al., 2009). The comparative cross-cultural analysis revealed that the native people in different ethnopharmacologies share extremely similar practices in the exploitation of medicinal insects, which are typically represented by hymenopteran and coleopteran insects (Costa-Neto, 2005; Dossey, 2010). The venom extracted from bees, wasps, and ants has been in use for treating autoimmune diseases such as rheumatism and arthritis in various ethnomedicines (Golden, 2006; Utkin, 2015), where bee products, including honey, royal jelly, propolis, and beeswax, are often used as folk remedies to treat wounds, infections, tuberculosis, cold, flu, sore throats, and other diseases (Münstedt and Bogdanov, 2009). Cantharidin is a defensive blistering agent secreted mainly by meloid and oedemerid beetles and is also found in hemipteran and dipteran insects (Young, 1984). This chemical was once well known for its abilities to boost sexual libido in European countries and was also used in various ways to treat rheumatism, anemia, carcinoma, and epidermal diseases in Egypt, Europe, East Asia, and probably in Latin America (Hemp and Dettner, 2001). To date, thousands of bioactive molecules have been identified from medicinal insects, and a few of them are *de novo* synthesized, structurally modified and even normalized to the popular pharmaceutical preparations, as represented by cantharidin, mellitin, solenopsin, silk nanofiber, pederin, etc. However, the medicinal role of the insect is still underestimated when compared to the widely prospected plant-derived pharmaceuticals. A previous report asserted that there are approximately 16 times as many insect species as there are plant species, while the knowledge depth and scope of phytochemistry far exceed those of insect chemistry when comparing the amount of research per species (Trowell, 2003). In addition to the crude insect drugs used in traditional ways, a large proportion of marketed entomoceuticals are now produced industrially, e.g., silk fibroins, bee products, melittin, and cordyceps. During the past 20 years, funding on entomoceutical research has grown rapidly with the involvement of known pharmaceutical companies and research organizations, such as Merck (Rahway, NJ, United States), Roche (Switzerland), BioValley (France), and Entocosm Pty. Ltd. (Australia), and the Instituto Nacional de Biodiversidad (Heredia, Costa Rica). Countries such as China and Korea have recently invested much in the varieties and market share of medicinal insects. Over the past 10 years, there has been a fast-growing trend in research and patents for entomoceuticals around the world.

Although the market potential of insect-derived drugs is highly expected, the current information available in bioprospecting medicinal insects from ethnopharmacologies remains incomplete. The recently published review articles focused on a few typical insect species and relevant medical applications, such as fruit flies, silkworms, ants, bees, wasps, blister beetles (Costa-Neto, 2002; Dossey, 2010; Dettner, 2011; Ratcliffe et al., 2011; Meyer-Rochow, 2017; Seabrooks and Hu, 2017; Dutta et al., 2019), and still a fair amount of entomoceuticals, especially from traditional Chinese medicine (TCM), provide minimal information for their varietal origins and medicinal applications (Jiang, 1999). It is worth noting that secretions or metabolites from insects are commonly produced with cascaded hormonal and metabolic regulation, but the process has seldom been mentioned in previous studies. This review first made a generalization for the currently used entomoceuticals based on their evolutionary status and highlighted 12 medicinal insect species in the traditional Chinese pharmacopeias that were well prospected for medical uses during the past 20 years. Additionally, we briefly specified the recent progress of the insect omics data (genome, transcriptome, metabolome, proteome) underlying these entomoceuticals. This text provides a supplement for insect folklore medicine and would be very valuable for novel drug discovery.

### Phylogenetic origin of medicinal insect species

**FIGURE 1:**
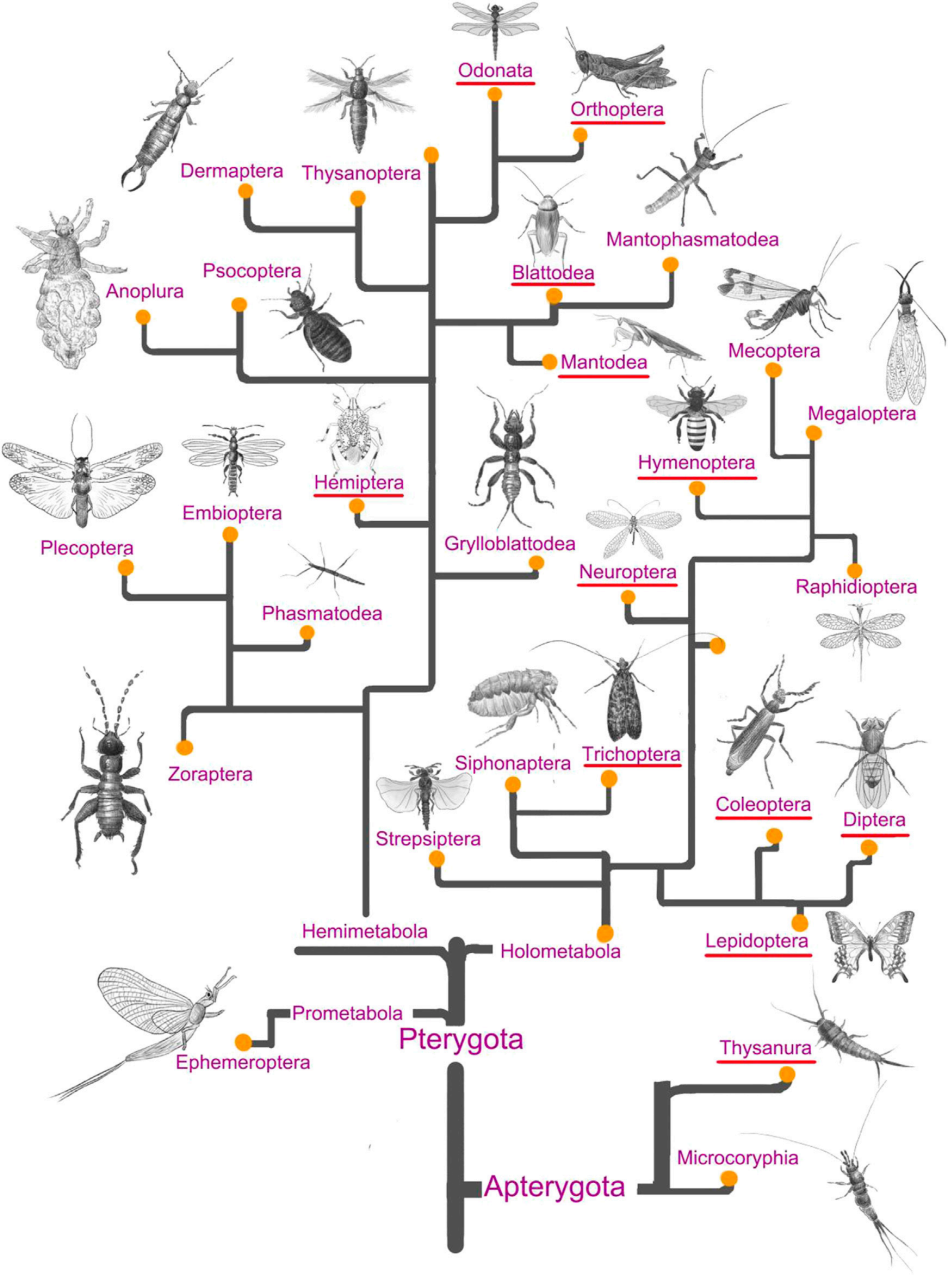
A total of 29 insect orders are graphically depicted according to the recently updated insect evolutionary system (Ishiwata et al., 2011; Misof et al., 2014). Twelve insect orders that have been exploited for drug discovery are characteristic of the red underline.

**FIGURE 2:**
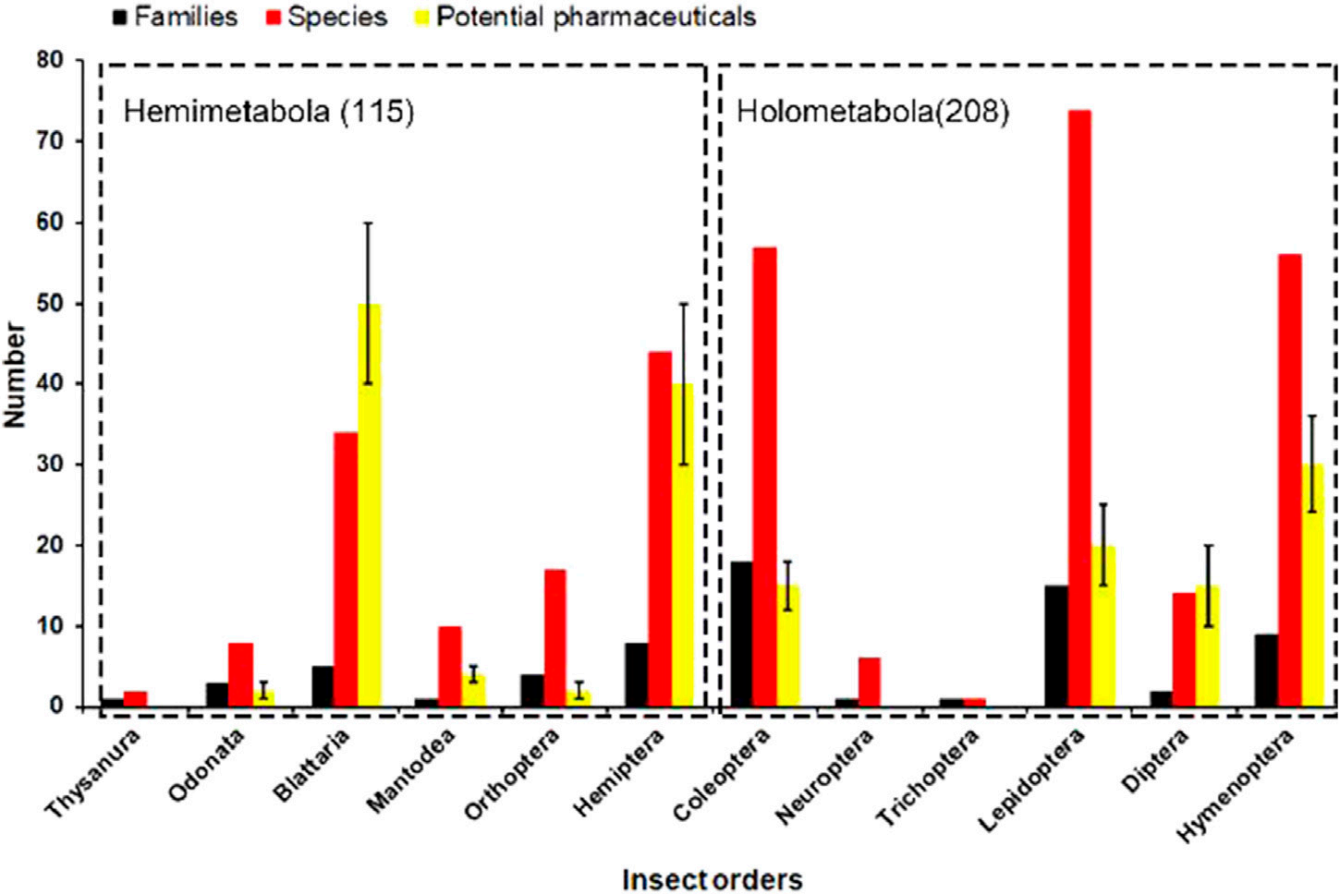
Phylogenetic analysis of medicinal insect species across 12 insect orders. The number of potential pharmaceuticals varies in most insect orders because some bioactive components remain to be validated for their pharmaceutical effects.

As shown in Figure 1, less than half of the total number of insect orders was exploited for medicinal purposes during the past, and 323 medicinal insect species were collected from the previous literature (Jiang, 1999; Commission, 2015). Hemimetabolous insects were intensively prospected for their potential pharmacological effects during the past decades, especially for pharmaceuticals from the orders Blattaria and Hemiptera (Figure 2). In addition, quite a few holometabolous insects from the same family (e.g., Hepialidae, Formicidae) were investigated for their endosymbionts (e.g., cordyceps, termitomyces) or glandular secretions (e.g., honey, venom), while the bioactive components isolated from their bodies were less prospected. It was estimated that 150–200 pharmaceutical agents were identified from the above medicinal insects, and their chemical structure and pharmacotherapies were well described and verified in previous studies.

### Medicinal insect species from TCM

**FIGURE 3:**
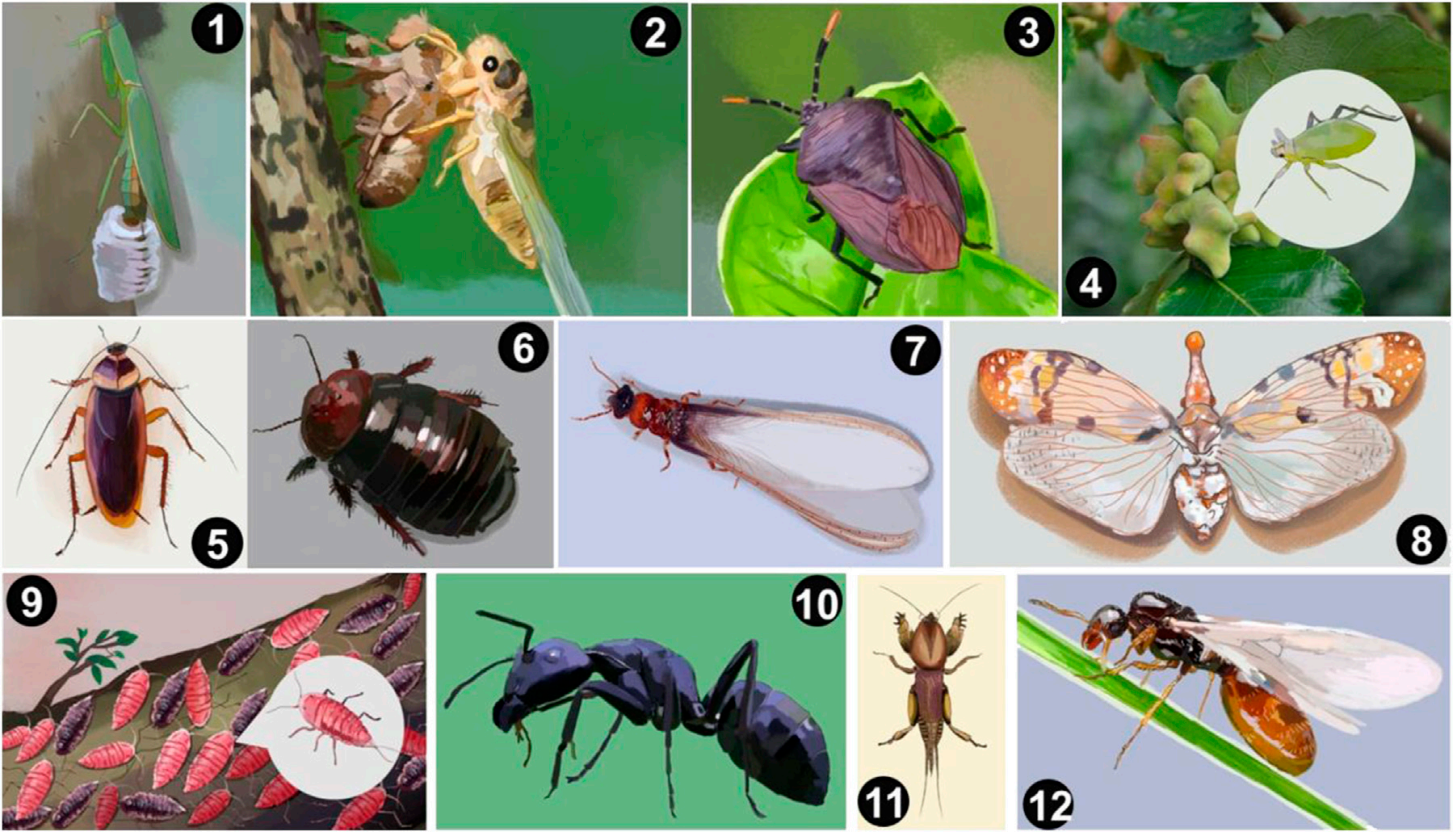
1A live portray of 12 insect-derived drugs from traditional Chinese medicinal herbals that have been widely explored for their active pharmaceutical agents over the past decades. (1) Mantis ootheca, (2) *Cicada* slough, (3) Stink bug, (4) Gallnut, (5) Cockroach, (6) Ground beetle, (7) Termite, (8) Wax scale insect, (9) Lac insect, (10) Black ant, (11) Mole Cricket, and (12) Gadfly.

The first Chinese physicians to apply medicinal insects to their practice date back to three thousand years ago, according to the earliest textual record indicated by the book ‘*Rites of the Zhou Dynasty* (*Zhou Li*)’, which was compiled officially during the West Han dynasty (202 BC-8 AD) and is the first reference to indicate crickets as a medicinal formula. The first Chinese medicinal monograph, ‘*Shennong’s Materia Medica Classic*’ (100-200 AD), described 22 kinds of insect-containing prescriptions, including white muscardine silkworm, honeybee, wasp, and mantis. Since then, the number of insect species used in herbal formulae has gradually increased with the subsequent publications of medical literature, such as the ‘*Compendium of Materia Medica’* (1552 AD-1578 AD), which is a giant encyclopedia of Chinese folk medicine that identified more than 70 medicinal insects. At least 11 ancient books of TCM were well passed down, and approximately 100 insect species and their medicinal applications were documented (Jiang, 1999). Namba et al. translated the knowledge of the medicinal insects that were recorded in the classic “herbal” Jing-shi-zheng-lei-da-guang-ben-cao (a.k.a. *Classified materia medica from historical classics for emergency*) edited during the Chinese Song dynasty (960-1280 A.D.) (Tsuneo et al., 1988). Today, more than 300 insects are filed in the Pharmacopoeia of the People’s Republic of China (Feng et al., 2009), but fewer than 20 medicinal insect species have been intensively prospected for their bioactive components using advanced techniques. In this overview, we highlighted 12 medicinal insect species (Figure 3) based on their chemical structures, pharmacological mechanisms, and pharmacokinetics.

### Mantis egg cases

Mantis egg cases (MECs) are the ootheca produced from preying female mantis, characteristically foam-like, long, and flat. The earliest use of MECs in TCM dates back to two thousand years ago and was well described in archaic Chinese medical literature such as ‘*Shennong’s Materia Medica Classic*’ and ‘*Mingyi Bielu*’ (456–536 AD). According to the newly published Chinese Pharmacopeia (Commission, 2015), at least three MEC types were prepared as medications according to the sources of the mantis species, which include *Tenodera sinensis*, *Hierodula patellifera*, *Stastilia maculate*, and *Mantis religiosa*. MECs not only have significant pharmacological effects, but also have high edible value. It was recorded in TCM that MECs were employed as diuretics to improve urinary dysfunction (Tan et al., 1997) and exploited as tonics for enhancing immunity. Jia et al. showed that MECs could rescue cyclophosphamide-induced immunosuppression in mice *by* promoting the proliferation and phagocytosis of macrophages, in accord with elevated immunoglobulins (IgM, IgG), cytokines (TNF-ɑ, IL-2, IL-4), and antioxidant enzymes (MDA, SOD, GSH-Px) (Jia et al., 2016). N-(3,4-Dihydroxyphenethyl) acetamide and 2,4-di-tert-butylphenol, two chemicals separated from MECs, were verified as strong antioxidants to scavenge DPPH radicals and were proven to effectively resist the oxidation of low-density lipoprotein (LDL) and apoB-100, thus being potent drug candidates against hyperlipidemia and atherosclerosis (Xu, 2014). Hahn et al. identified two enzyme genes, mantis egg fibrolase (MEF) and mantis egg fibrinolytic enzyme (MEF-2), both of which were specific to serine proteases, and specifically showed high activity toward benzoyl-Phe-Val-Arg-*p*-nitroanilide, a substrate for thrombin and trypsin, respectively as 10.56 U/mg and 155 U/mg (Hahn et al., 1999; Hahn et al., 2001). In addition, it was reported that the extracted oil substances from the mantis ootheca were effective in inhibiting the growth of infectious bacteria, e.g., *Staphylococcus aureus* (Yira, 2014) and *Pseudomonas aeruginosa* (Wang et al., 2018) with a minimum inhibiton concentration (MIC) of 4 mg/mL.

### *Cicada* slough

*Cicada* slough (CS), also called Periostracum cicadae, is translucent and luster shelled, usually obtained from the trunk at the end of the molting of the cicada larvae. In TCM, *Cryptotympana pustulata* Fabricius (Hemiptera: Cicadidae) is officially claimed to be the *bona fide* origin of CS, and this insect is typically of dark color and widely distributed in China. There have also been other cicada species that are considered sources of exuviae in the Chinese herbal market, including *Auritibicen flammatus*, *Cryptotympana mandrina*, and *Platypleura kaempferi* (Jiang, 1999). CS has often been used as a Chinese folk medicine to improve throat discomfort, relieve spasms and defervescence, treat skin diseases, and combat allergies and sphagitis. The immunomodulatory effect of CS was observed by Xu et al. (2006) using an asthma model of guinea pigs, in which the pathological conditions of the lung and the inner bronchia were well remedied when treated with the water extract of CS, while the levels of IL-2, IL-5, TXB2, and 6-Keto-PGF1αin serum recovered to the normal state (Zhang, 2007). In addition, a small peptide (F2-2-2) isolated from CS was found to be a potential fibrinolytic compound that effectively decomposed the blood stasis and dissolved the aggregated platelets, but its protein structure remains to be investigated. At the same time, CS can also be used in cooking and it has good therapeutic value (Melgar-Lalanne et al., 2019).

### Stink bug

The stink bug (*Aspongopus chinensis* Dallas) belongs to the hemimetabolous insect order (Hemiptera: Pentatomidae) and is known for the fetor emitted from its specialized gland that is used as a chemical odor against dangers. *A. chinensis* D. (ACD) naturally resides in China and is usually found to feed on cucurbitaceous crops, leading to serious economic losses to farmers. However, natives in southwest China have exploited this bug for its nutritional benefits, e.g., fat acids, proteins, amino acids, vitamins, and trace elements (Liu and Jian-Ping, 2008; Li and Li, 2010). The crude oil analysis revealed a high level of unsaturated fatty acids (FAs) in ACD, accounting for approximately 60% of the total FAs, of which oleic acid occupies the largest proportion, varying from 44% to 50% (Li S. et al., 2020). ACD has been recorded as the herbal medicine in TMC that is primarily applied for treating sexual dysfunction and tumors and has also been reported to have many other pharmacological activities, such as treating kidney diseases, removing blood stasis, antioxidant, anti-inflammation, and anti-fatigue activities. It was shown that mice undergoing reproductive damage when exposed to manganese were partly rescued by this bug, during which the antioxidant enzymes and the anti-apoptotic genes from the Bcl-2 family were highly activated, and the expression levels of the (pro-) apoptotic genes Bax, CytC, and cleaved Casp 3 were significantly inhibited (He et al., 2016; Jiang et al., 2019; Liu Q. et al., 2019). Several non-peptide small molecules have been further identified as potential drug candidates for improving renal fibrosis and inhibiting extracellular matrix expression in mesangial cells under diabetic conditions, as well as potent inhibitors of Smad3 phosphorylation (Shi et al., 2014; Yan et al., 2014; Di et al., 2015; Liao et al., 2020). However, only (±)-Aspongopusamide A, Aspongopusamide C, and Aspongamides C were investigated for their inhibitory effects on COX-2, and their IC_50_ values were 6.5, 117, and 6.86 µM, respectively. The hemolymph or the crude extract of this bug was proven to effectively inhibit the proliferation of cancer cells (e.g., HepG2, MCF-7, SGC-7901) and the ensuing (pro-) apoptosis *via* cell cycle arrest (G_0_/G_1_ phase) and the regulation of signaling pathways such as STAT3/Survivin/Bcl (Hou et al., 2012; Shen, 2015; Yang et al., 2017; Wang and Wang, 2018; Wu Y. F. et al., 2018). An anticancer active mixture separated from the bug haemolymph was identified to be composed of 18 protein families, including cytochrome c, ferritin, superoxide dismutase, and hemocyanin-like protein. All of which might jointly inhibit cancer cell growth (SGC-7901: IC_50_ = 25.462 μg/mL and BGC-823: IC_50_ = 29.003 μg/mL) and partially suppress tumor growth in a 4T1 xenograft mouse model (Tan et al., 2019).

### Chinese gallnut (CG)

Chinese gallnuts or nutgalls (Galla Chinensis) are overgrown plant tissues induced by invading larvae of gall insects that are mainly derived from two insect species. One is the aphid from the family Pemphigidae, which infests the compound leaves of *Rhus* spp. and results in deformed bulbs, and the other is a parasitic wasp from the family Cynipidae, which parasitizes the fresh branches or twigs of *Quercus infectoria* Oliver and produces aberrant protrusion (Djakpo and Yao, 2010; Sariozlu and Kivanc, 2011). The earliest medicinal use of CGs was recorded in the ‘*Compendium of Materia Medica’* and its remedial effects on tuberculosis, hemostasis, diarrhea, insomnia, etc. have been well described in TCM. China has occupied the major market share (>90%) of gallnuts and relevant products globally. To date, at least sixteen species of gall-forming sumac aphids have been identified (Zha et al., 2014). There have been classifications of fourteen types of gallnuts that are differentiated by their parasitic aphid species and further generalized into three large groups based on their number of gallnuts on compound leaves, the content of tannin, water, and other constituents in the gallnut: they consist of belly shaped gallnut, horned gallnut, and inflorescence gallnut (Yang et al., 2008). Gallnuts are involved in a variety of industrial products, including dyestuff, tanning agents, food preservatives, oil-soluble antioxidants, clarifying agents, and pharmaceutical raw materials. Gallic acid, ellagic acid, gallotannins, phenolic acids, triterpenoids, and flavonoids were identified from the extract of gallnut, and gallotannins account for more than 50% of the dry weight (Gu, 2012; Yang et al., 2016). Gallotannin is a compound mixture mainly composed of penta–dodeca-galloylglucoses, which have a ‘penta-O-galloyl-β-D-glucose’ core attached with a varying number of galloyl group(s) at its C-2, C-3, and C-4 position(s) (Niemetz and Gross, 2005). CGs have been reported to be potent antioxidants for DPPH radicals (EC_50_ = 1.22 ± 0.01 μg/mL in ethyl acetate, IC_50_ = 13.13 μg/mL in water) (Tian et al., 2009; Park et al., 2019), and the antioxidant effects of CGs mainly originate from gallotannin and gallic acid. CGs have been employed for antithrombosis (Song et al., 2002), antiaging (Li et al., 1999), alleviating radiation-induced damage (Sun et al., 2019), and protecting teeth by orally inhibiting cariogenic bacteria, such as *Streptococcus mutans* UA 519, *Actinomyces viscosus*, and *Enterococcus faecalis* (Liu and Xu, 2017). *In vitro* bioassays have demonstrated that water extracts of Chinese gallnuts effectively protect periodontal ligament cells and enhance the remineralization of initial artificial enamel lesions (Wang et al., 2005; Zhang et al., 2011; Wang et al., 2013). In China, CG products have been processed as a major component in medical toothpastes that are very popular in supermarkets. Gallic acid is the core representative of phenolic acids, accounting for approximately 2%–4% of the dry weight of Chinese gallnut, and this chemical has been widely studied for its antitumor and antiviral effects. In addition, other potential anti-inflammatory and antitumor compounds from gallnut have been identified, such as syringic acid, ellagic acid, methyl gallate, *β*-sitosteriol, purpurogallin, and amentoflavone (Wang et al., 2005; Wang et al., 2013). CG has a variety of pharmacological activities, and its toxicity is low, so it can be widely used in medicine, food and other fields.

### Cockroach, ground beetle and termite

The cockroach (Blattidae) is a notorious hygiene pest that pesters people with its powerful vitality, ubiquitous distribution, and behaviors, such as preferring rotten foods, carrying numerous infectious pathogens that often cause intestinal diseases and arouse allergic reactions in hypersensitive populations (Mullins, 2015). However, this pest has been used medicinally by ancient Chinese people for more than two thousand years, as recorded in ‘*Shennong’s Materia Medica Classic*’. The American cockroach (*Periplaneta americana* L.) is among the most studied cockroach species for its medicinal value. China has now developed the largest rearing industry of *P. americana* to meet the pharmaceutical market, and dozens of tons of cockroaches are produced every year (Xie et al., 2018). In TCM, *P. americana* has often been mixed with *Blatta orientalis* L. to be medically used, and some studies have suggested that *B. orientalis* has the same pharmaceutical activities as *P. americana*. At least three cockroach-derived drugs have been approved for clinical therapy in China: ‘Kangfuxin’ (KFX), ‘Xinmailong’ (XML), and ‘Ganlong Capsule’ (GLC) (Zhang et al., 2017; Lai et al., 2019; Wang et al., 2019a). KFX is produced from the ethanol extract of *P. americana* that has been employed for treating chemotherapeutically induced mucositis and gastrointestinal ulcers (Luo et al., 2016; Zou et al., 2019). XML is a complex group of nucleotides and amino acids separated from *P. americana* that is mainly applied for improving chronic heart failure (Lu et al., 2018), at least four active molecules isolated from XML were found to be related to antithrombosis (e.g., Adenosine, Protocatechuic acid, Inosine, Pyroglutamate dipeptides). GLC is composed of compounds extracted from *P. americana*, *Bupleurum* sp., *Scutellaria barbara*, dandelion, and other medicinal herbals, which have proven therapeutically effective on hepatic fibrosis (Chen et al., 2019). The potent antioxidant activities of both drugs and activation of cytokines (e.g., TNF-ɑ, IL-6) and MAPK-associated signaling pathways (e.g., Syk/PLCγ2, PI3K/Akt/GSK3β) underlying the above remedying effects were well revealed in previous studies using mouse models (Ma et al., 2018a; Lu et al., 2019; Wang et al., 2019b). KFX was also reported to exert proapoptotic and antiosteoporosis effects on human stomach cancer cells (SGC-7901) and bone marrow mesenchymal stem cells (BMSCs) *by* triggering the ERK/p53 apoptotic pathway and increasing the secretion of osteocalcin and mineralization of osteoblasts, respectively (Huang et al., 2017; Ma et al., 2018b). An increasing number of active compounds related to the therapeutic effects of *P. americana* have been identified in recent years, and at least four active molecules isolated from ‘Xinmailong’ were found to be related to antithrombosis (Qi et al., 2017). In addition, multiple active components from *P. americana* have been identified as potential drug candidates for wound healing and anti-fungal therapy (Yun et al., 2017; Fang et al., 2018; Zhu et al., 2018; Yan et al., 2019). D-glucosamine and its salts, which are obtained from cockroaches, are employed in dietary supplement products, which have demonstrated positive effects on the treatment of osteoarthritis, knee, and back pain and has an important role in the formation of joints, sinews, bones, heart valves, and respiratory tract (Bertuzzi et al., 2018). The cockroach, as an edible insect, is rich in protein and has a high nutritional value, so it can be widely used in the food field (Melgar-Lalanne et al., 2019).

The ground beetle is morphologically similar to the cockroach, and both belong to the insect order Blattaria. Comparatively, this insect is less appalling to people and feeds less on decaying food (Hu et al., 2011). In some countries, such as China and Thailand, the ground beetle has been officially recommended as a healthy food with high contents of proteins (21–54 g/100 g dry weight), lipids (33.40 g/100 g dry weight), essential fatty acids, vitamins, and a variety of beneficial minerals (1–7 g/100 g dry weight) (Verkerk et al., 2007; Chen et al., 2009). In China, at least four species of ground beetles that belong to the family Corydiidae are compiled into traditional Chinese medicinal prescriptions, including *Eupolyphaga sinensis* Walker (ESW), *E. thibetana*, *E. everestians*, and *Polyphaga plancyi*, among which ESW is the most widely exploited. In the formula preparation, ESW is usually dried and ground into powder to be used together with other herbal drugs and used as a whole for tinctures. It has been recorded in the Chinese pharmacopeias that ESW has been widely used to treat blood stasis, cancers, septic joints, rheumatoid arthritis, herpes zoste, and hyperostosis. ESW contains multiple pharmacologically active substances that have been identified as antioxidants, AMPs, thrombolytic agents, vasodilators, angiogenesis potentiators and protease inhibitors of cancer cell proliferation. At least four low molecular weight components were isolated from ESW to be used as drugs for cancer treatment, anti-hyperlipidaemia, anti-inflammation, and sedation (Yang and Liu, 2015). Recent studies have shown that antioxidant peptides isolated from ESW have strong protective effects on mouse livers and can effectively alleviate UV radiation-induced skin photoaging by activating antioxidant enzymes (e.g., SOD, CAT, GPH-Px) and associated Nrf2/ARE signaling pathways (Zhang et al., 2019). A termicin-like peptide separated from ESW (Es-termicin) shows very high similarity with the amino acids of termicin in dipteran insects, exhibiting strong inhibitory effects on *Candida albicans* ATCC 90028 (Liu Z. et al., 2016). An early *in vivo* study showed that ESW ethanol extract (ESWE) treatment could effectively promote the apoptosis of hepatocarcinoma cells in an H_22_ xenograft mouse model *via* the upregulation of Th1-type cytokines (TNF-ɑ and IFN-γ) and activation of Bcl-2 and caspase family members (Ge et al., 2012). Dai et al. revealed that ESWE (70% ethanol extract of ESW) exhibited inhibitory effects on the proliferation of A549 cells *via* antiangiogenic activity and interrupting the autophosphorylation of the KDR signaling cascade (Dai et al., 2014). There is evidence suggesting that ESWE inhibits the proliferation of human hepatocellular carcinoma (HHC) cell lines *via* the downregulation of the canonical PI3K/AKT/mTOR and c-Jun N-terminal kinase signaling (MAPK) pathways, as well as their downstream effector molecules, such as MMP2, MMP9, and CXCR4 (Zhang Y. et al., 2014). Similar results were observed in ESWE-treated breast cancers (i.e. MDA-MB-435s and MDA-MB-231), the growth and metastatic activities of both cancer cell lines were significantly suppressed by ESWE through the downregulation of ERK1/2 and downstream signaling molecules, such as CXCR4, MMP2, and MMP9, and tumor growth in the MDA-MB-231 xenograft mouse model was notably inhibited by ESWE (Zhan et al., 2016). A potent antitumor protein EPS72 (MW: ∼72 kDa) isolated from ESW was found to induce A549 cell apoptosis with a low IC_50_ value, inhibit cell adhesion to fibronectin and collagen IV, and restrain cell migration and invasion (Wang et al., 2013). Feeding experiments demonstrated that the addition of EES to feedstuffs could improve the exercise performance and the activities of antioxidant enzymes in the skeletal muscle of rats (Gao et al., 2015), enhance immune function *by* promoting the serum NO content and triggering the activities of acid phosphatase and alkaline phosphatase, and reverse immunosuppression in cyclophosphamide (CTX)-treated mice (Tang, 2011; Liu H. et al., 2019). Over the past 20 years, studies about the isolation and identification of antithrombotic substances from EES have advanced rapidly. An early study successfully identified two antithrombin-like peptides (ET-I and ET-II) from ESW homogenates, all of which belonged to the serine proteinase family and exhibited strong fibrinolytic activities on *in vitro* fibrin plates, and the amino acid sequences of ET-II (MW: 32.9 kDa) were found to be orthologous to Homo plasma kallikrein B1 (40%), the fibrinolytic enzymes of *Scolopendra subspinipes mutilans* (45%) and *Lumbricus rubellus* (35%) (Li, 2006). A bifunctional protein (eupolytin 1, MW: ∼26 kDa) containing both fibrinolytic and plasminogen-activating (PA) activities was identified from ESW and showed potent and rapid thrombolytic ability and safety *in vivo* (Yang et al., 2011). Wang et al. employed proteomic and transcriptomic analysis to reveal 105 serine proteases belonging to four families (families 1–4) that are potential fibrinogenolytic candidates (Wang et al., 2012).

The termite is a well-known cosmopolitan pest that usually has destructive effects on agricultural crops and dwelling houses and even breaks down the foundation of dams and results in serious floods (Ghaly and Edwards, 2011). The termite is the only hemimetabolous social insect with nest-building behavior that is similar to the ant but is evolutionarily related to the cockroach and has been reclassified from the order Isoptera into the order Blattidae (Inward et al., 2007). This insect was also recorded as a medical formula in the ‘*Compendium of Materia Medica*’, and at least three winged termite species have been prescribed into traditional Chinese herbal medicines, including *Coptotermes formosanus* (Family: Rhinotermitidae), *Reticulitermes speratus* (Family: Rhinotermitidae), and *Macrotermes annandalei* (Family: Termitidae). Termites were found to contain high levels of proteins, essential amino acids, unsaturated fat acids, sterols, vitamins, and especially abundant iron content (Li, 2002; Cu et al., 2012). Termites have been consumed as an alternative food by humans for thousands of years, and some tribes in Asia, Africa, Australia, and South America still have the habit of eating termites through direct chewing, frying, drying, and roasting (DeFoliart, 2009). In China, termites have been processed into a daily tonic by means of dehydration, alcoholic fermentation, and packed capsulation, which has been approved by regulatory agencies for sale (Yan et al., 2008). The medical uses of termites in various folk medicines were well summarized in a previous report, including the treatment of asthma, bronchitis, influenza, sore throat, sinusitis, rheumatism, child malnutrition, fatigue, etc. (de Figueiredo et al., 2015). An early exploration revealed that the water extract of termites could inhibit HIV-1 viral activities, implying that potential drug candidates could be screened out from termites for HIV-1 therapy (Lin et al., 2008). In recent years, more studies have focused on the investigation of the pharmaceutical activities of the termitarium, a fungus comb built from termite faecal pellets with partially digested plant debris (Hyodo et al., 2003). Fungus-growing termites usually live in a symbiotic relationship with the genus *Termitomyces* (Basidiomycota, Lyophyllaceae), which serves as a protein-rich food source and an auxiliary sink to decompose lignocellulose uptake by termites (van de Peppel and Aanen, 2020). In TCM, termitarium was reported to have many therapeutic effects, including immuno-enhancing effects, anti-cancer, anti-inflammation, ageing resistance, sexual enhancement and neuroprotection. Previous evidence-based analysis demonstrated that the termite nest contained large amounts of pharmaceutical agents, e.g., polysaccharides, sesquiterpenoids, cerebrosides, heteroglycans, isoflavonoids, etc. (Hua et al., 2015; Lee et al., 2018; Li et al., 2019; Zhao et al., 2019), most of which were well summarized in a previous review (Hsieh and Ju, 2018). Herein, we only compiled a general overview of the newly found active compounds from *Termitomyces* sp. from 2018 to 2020. The termite mushroom (*Termitomyces albuminosus*) is found to be among the more dominant fungal species growing near the termitarium, which has been domesticated for food and drug resources (Qian, 2011). Recent research first suggested that *Termitomyces albuminosus* powder (TAP) is safe for human consumption (Park et al., 2021). It is now generally believed that the pharmacological effects of *T. albuminosus* are equal to those of termites and termitaria (Wang, 2012).

### Insect wax and shellac

These two substances are the glandular waxy secretions of coccid insects from the *Coccoidae* family and are primarily applied to industrial raw materials. Insect wax is secreted by *Ericerus pela* that lives off of the Chinese ash (*Fraxinus chinensis*) and the privet (*Ligustrum lucidum*). As a edible insects for both food and medicine, Insect wax has been widely used as fuel, moulding, lubricants, polishing compounds, health supplements, cosmetics, tablet coatings, etc. And its medication roles in hemostasis, pain relief, wound healing, tissue regeneration, and neuroleptics have been well described in TCM. A recent study showed that insect wax is an ideal inducer to promote hair growth by upregulating vascular endothelial growth factor (VEGF) expression (Ma J. et al., 2018). Another study showed that policosanol derived from insect wax (PIW) may be a potential therapeutic agent for the prevention and treatment of Alzheimer’s disease (AD) (Zhang et al., 2021). Shellac is a natural polymer resin derived from lac insects (*Kerria* spp.) that infest more than 300 plant species. Similar to the commercial use of insect wax, shellac is extensively used in polishing, varnish, molding, glue, and coatings for sugar and tablets. In traditional Chinese medicine, shellac has been prescribed for treating hemorrhage, measles, macula and scabies. The major pharmaceutical agents identified from shellac are a collection of sesquiterpene acids, during which only shellolic acid A wasproven to have antimicrobial activity against *B. subtilis* (MIC = 0.1 mg/mL) (Lu et al., 2014). Today, shellac is a major constituent for enteric-coated capsules to deliver drugs for medical purposes.

### Black ant

As mentioned earlier, the venom secreted by the ant and its endosymbiont-derived chemicals are potential pharmaceuticals. Ants, rich in protein and unsaturated fatty acids, are consumed in many regions of the world, particularly in southern Asia (Feng et al., 2018). In addition, the whole body of the ant has been exploited in traditional Chinese medicinal prescriptions to treat inflammation, hyperuricemia, cancer, rheumatoid arthritis, depression, fatigue, insomnia, itching, epilepsy, etc. In China, *Polyrhachis vicina* Rodger is among the most widely distributed and exploited ant species with the name ‘*black ant’* owing to its typical black body. The black ant is a safe edible insect containing appreciable amounts of proteins that has been recommended as a healthy food for enhancing immunity, resisting aging, and improving sexual dysfunction in the Chinese health-preserving culture (Wang and Wang, 2010). Processed black ants undergo various processes, including dehydration, packed capsulation, and alcoholic fermentation, and are massively produced for non-prescription health products in China, South Korea, Japan, and other countries (Liu et al., 2006). It was reported that the active fractions from the black ant are composed of dopamine, alkaloids, amino acids, nucleotides, fatty acids, cyclopeptides, triterpenoids, simple phenolic compounds, etc., some of which have been recently isolated, purified, and validated for their pharmaceutical activities (Zheng et al., 2012). For instance, the fatty acid obtained from the dried black ant has shown potential as an antioxidant, metabolic regulator, and immunomodulator and recently has been employed for treating hyperuricemia, depression, and breast cancer (Hui et al., 2008). GC-MS analysis revealed that unsaturated fatty acids accounted for more than 70% of the total fatty acids in the black ant, including octadacenoic acid, heptadecenoic acid, and leinoleic acid (Oranut et al., 2010; Liu J. et al., 2016). A recent study extracted an active fraction from the black ant, identifying it to contain more than 70% unsaturated fatty acids, which was proven to be therapeutically effective in suppressing cell proliferation, migration, and invasion and inhibiting tumor *growth* (MCF-7: IC_50_ = 17.91 mg/mL; MDA-MB-231: IC_50_ = 18.73 mg/mL) *by* regulating the canonical EGR1/NKILA/NF-κB axis (Li D. M. et al., 2020).

### Gadfly

The gadfly is a general name for the insect species belonging to the family Tabanidae, and at least 27 Tabanidae species affiliated with three genera (i.e. *Pangonlinae*, *Chrysopsinae*, *Tabaninae*) were medicinally used in TCM (Jun-De et al., 2010). The gadfly is well known for its strong blood-sucking capability by piercing into the pachyderms, sometimes even feeding on human blood. The earliest recorded use of these insects as medicinal herbs was in the book ‘*Shennong’s Materia Medica Classic’*. The gadfly was mainly employed by traditional medicinal practitioners as an anticoagulation agent and applied for treating coronary heart disease, stroke, headache, liver cirrhosis, psoriasis, and hepatic carcinoma (Committee, 2015). The horse fly (*Tabanus* sp.) is among the most explored genera in the family Tabanidae for its active pharmaceutical agents, including polypeptides, fatty acids, glycoproteins, neuropeptides, and a variety of trace minerals. During the past few decades, the salivary gland of the gadfly has become the research focus for the discovery of novel peptides for treating blood stasis. At least five antithrombotic proteins were successfully isolated and identified from the gland. Thrombostasin (Zhang et al., 2002), TAP (Ahn et al., 2006), Tablysin-15 (Ma et al., 2011), vasotab TY (Zhang Z. et al., 2014), and Tablysin 2 (Sheng et al., 2017). Two immunoregulatory peptides, TP1-3 (Zhao et al., 2009) and cecropin-TY1 (Wei L. et al., 2015), separated from the salivary gland of the horsefly were found to suppress the lipopolysaccharide (LPS)-stimulated cytokine storm *via* the inhibition of intereron-γ (INF-γ), monocyte chemoattractant protein (MCP-1), and nitric oxide (NO) and thus are potential anti-inflammatory drugs. The salivary gland extracts of the horsefly were also reported to contain potent vasodilators that could effectively reduce left ventricular pressure (LVP) (Rajska et al., 2007). Except for the species in *Tabanus* sp., Chrysoptin, a potent antagonist for the fibrinogen receptor was identified from the deerfly (genus *Chrysops*) and was found to be effective in inhibiting ADP-induced platelet aggregation (Reddy et al., 2000). Here, we only indicated fibrinolytic peptides from *Tabanus* sp. or other blood-sucking flies whose sequences were wholly or partly identified. There were other reports on antithrombotic proteins but with only an estimate of molecular weight in previous studies (Chen et al., 1982; Park et al., 1998; Yang et al., 1998; Yang et al., 1999; Yang et al., 2000). Whether these early findings are novel or identical to the known sequences remains to be investigated.

### Mole cricket

This insect has historically been regarded as a major underground pest in China that could destroy the roots and seedlings of agricultural crops, leading to heavy losses to farms. It was reported that there are at least five species of mole crickets widely distributed in China, among which *Gryllotalpa unispina* Saussure and *Gryllotalpa orientalis* Burmeister have often been exploited in traditional Chinese herbal medicine for the treatment of edema, urinary disorders, toothache, etc. The mole cricket was reported to contain high levels of proteins, fatty acids, and free amino acids, with medicinal amino acids accounting for more than 50% of the total amino acids (Guo and Wei, 2006; Sun et al., 2011). The insect has been massively reared as an important food supply for livestock and has been attested to be an alternative growth promoter without chronic toxicity. Four ingredients identified from the methanol extract of *G.* orientalis were validated to be antimicrobial, including berberine, palmatine, two *Buxus* alkaloids (*Gryllotalpa A* and *Gryllotalpa B*) (Niu 2015; Liu, He, et al., 2019). A recent study provided evidence regarding the similarity between water extracts of *G. orientalis* and hydrochlorothiazide on saline-loaded mice in its diuretic effect but also showed that they share differences in the modes of function on the excretion of potassiumions (Wei X. et al., 2015). Zi et al. found that the ethanol extract of *G. orientalis* showed significant cytotoxic effects on three human cervical cancer cell lines (HeLa, Caski, and C-33A) (Mi et al., 2000; Jiaji et al., 2017). Furthermore, *G. orientalis* extracts acquired from various organic solvents were strong candidates to clear off free radicals efficiently and significantly repress COX-2 promoter activities, thus being ideal antioxidants for oxidative stress-induced lesions (Heo et al., 2008). Mole cricket not only does not have toxicity, and have diuretic, sedative and other effects, and is constantly developed into various types of nutrition and health food (DeFoliart, 2002).

### Insect omics in relation to entomoceuticals

In the last 10 years, high-throughput screening methods using multiomics (i.e. transcriptomics, proteomics, microbiomes, and metabolomes) to mine potential drugs or drug-associated synthetic routes from insects sped up the discovery of novel drug candidates. For instance, cantharidin was *de novo* synthesized from farnesol in the meloid beetle, in which the mevalnoate (MVA) and methylerythritol 4-phosphate/deoxyxylulose 5-phosphate (MEP/DOXP) pathways were involved, and three genes (i.e. *McMenA*, *FPPS*, and *HMGR*) were found in the blister beetle to be key enzymes for cantharidin synthesis using conventional molecular cloning methods in previous studies (Lu et al., 2016; Zha et al., 2017; Liao et al., 2020). A recent transcriptomic analysis screened at least 14 candidate genes, including the above three that are likely implicated in cantharidin synthesis, and suggested that cantharidin biosynthesis in the blister beetle might only occur *via the* MVA pathway, independent of the MEP/DOXP pathway (Huang et al., 2016). Thereafter, two draft genomes of two blister beetles, *Hycleus cichorii* and *H. phaleratus,* confirmed the previous transcriptomic data and verified the MVA pathway as the only route for cantharidin synthesis (Wu Y. M. et al., 2018). Generally, from the first announcement of the fruit fly *D. melanogaster* genome during the 2000s to the kick start of the i5K Pilot Project (‘i5K’ is an initiative to sequence the genomes of 5,000 insects and other arthropods) (Levine, 2011), more than 400 insect species have been sequenced and assembled, and their genome drafts have been issued online and continuously updated (http://i5k.github.io/arthropod_genomes_at_ncbi). Approximately 50 insect species that are related to medicinal use have been sequenced and uploaded to the ‘i5K’ genome database, whereby the genetic information available from multiomics analysis can be referred to by developers to optimize key gene clusters governing the synthetic route of bioactive molecules, which can be remodeled using *in vitro* cell or microbial cultural systems and facilitate further breeding of medicinal insects with superior traits in producing potential pharmaceutical agents. Insect venomics is another emerging research topic in recent years; at least 14 lineages of venomous insects belonging to six orders have been classified, but Hymenoptera is the only source of venomous insects in which detailed structural and functional characterization of toxins has been applied to more than a few species (Walker et al., 2018). From the beginning of 2010, a consortium of academic and industrial partners from five European countries launched the FP7 Venomics Project (Genoscope), aiming at establishing a library for venomics, involving thousands of bioactive molecules that can serve as lead reservoirs for drug development in relation to human health, and the hymenopteran venomics from honeybees and parasitoid wasps were highlighted in the project (Gilles and Servent, 2014). Except for the early findings of the venomic composition (e.g., melittin, phospholipases, serine protease, antigen 5), an increasing stream of novel peptides and active chemicals were identified from the hymenopteran library for venomics, often applying integrated analysis of the proteome and transcriptome (Santos et al., 2011; Moreau and Asgari, 2015; Walker et al., 2018; Wilson and Daly, 2018; Senji Laxme et al., 2019). Therefore, the sequence and metabolic data excavated from insect venomics have greatly improved the realm of insects, which lags behind the findings of venomics from snakes, scorpions, spiders, centipedes, cone shells, and anemones. More importantly, compared to other venomic animals, insect venoms show more advantages in terms of cost, sustainability and biosafety for new drug development.

## Conclusion and perspective

Traditional Chinese medicine (TCM), among ethnomedicines, has the most recorded numbers of medicinal insects, with more than 300 species covering 13 orders (Feng et al., 2009), far outnumbering the total number of insect species collected in the other ethnopharmacologies. From the beginning of the 21st century, insect-derived drugs have garnered much interest in pharmaceutical research with an increased emphasis on naturally extracted drug discovery and evidence-based efficacy for various ailments, *viz.* wound healing, anti-inflammation, anti-cancer, anti-angiogenesis, anti-coagulation, and especially chronic diseases. Nevertheless, the often used medicinal insects, such as silkworm, bee, wasp, cockroach, and blister beetle, and most of the insect-derived drugs that were recorded in the ancient medical literature remain underexplored for their bioactive ingredients. In this review, we emphasized 12 medicinal insects from traditional Chinese prescriptions and their pharmaceutical agents and medicinal applications, which have been relatively extensively investigated during the past decade. Interestingly, more than half of the investigated medicinal insects, including cockroaches, black ants, termites, mole crickets, gall aphids, and coccids, are known pests that have been extensively studied for their management and control in agriculture. Thus, turning these ‘agricultural/urban pests’ into ‘medicinal treasures’, rather than simply eradicating them, has become a unique industry in China that can improve the economic wellbeing of low-income famers. Notably, although most of the newly identified entomoceuticals were verified well in their therapeutic efficacy for certain diseases, the relevant toxicological effects have been less explored in previous studies. As such, more *in vitro*/*in vivo* toxicity assays should be required before the approval of these insect-derived drugs for clinical trials. Furthermore, with the introduction of cutting-edge analytic tools (i.e. insect omics) into the entomoceutical research, more potential entomoceutical candidates are promised to be screened in the future.
