# Supplementary material for: A minireview of the medicinal and edible insects from the traditional Chinese medicine (TCM)
Source: Front Pharmacol. 2023 Mar 16;14:1125600. doi: 10.3389/fphar.2023.1125600 (PMC10060509; doi:10.3389/fphar.2023.1125600)
Supplement: Supplementary file 1 [file Table1.DOCX]

**Table S1. Phylogenetic status of medicinal insects (323) and their active pharmaceutical agents**

| Evolutionary status | | Insect order | Family | Species | `Representative pharmaceutical agents | | |
| --- | --- | --- | --- | --- | --- | --- | --- |
|  |  |  |  |  | Number/Name | | References/Origin |
| **Apterygota** | | Thysanura | Lepismatidae | *Ctenolepisma villosa* | Unknown | | |
|  |  |  |  | *Lepisma saccharina* |  |  |  |
| **Pterygota**  **Pterygota**  **Pterygota**  **Pterygota**  **Pterygota**  **Pterygota**  **Pterygota**  **Pterygota**  **Pterygota**  **Pterygota**  **Pterygota**  **Pterygota**  **Pterygota** | **Hemimetabola**  **Hemimetabola**  **Hemimetabola**  **Hemimetabola** | Odonata | Coenagrionidae | *Coenagrion* sp. | amicoumacin B  (LD50: 132 mg/kg mouse, perorally) | | (Thongtan et al., 2002)  endosymbionts |
|  |  |  | Libellulidae | *Crocothemis servilia* |  |  |  |
|  |  |  |  | *Pantala flavescens* |  |  |  |
|  |  |  |  | *Sympetrum darwinianum* |  |  |  |
|  |  |  |  | *S. infuscatum* |  |  |  |
|  |  |  |  | *Orthetrum melania* |  |  |  |
|  |  |  | Aeschnidae | *Aeschna melanictera* |  |  |  |
|  |  |  |  | *Anax parthenope julius* |  |  |  |
|  |  | Blattaria | Blattidae | *Periplaneta americana* | See the article | | endogenous/ endosymbionts |
|  |  |  |  | *P. australasiae* |  |  |  |
|  |  |  |  | *P. fuliginosa* |  |  |  |
|  |  |  |  | *Blatta orientalis* |  |  |  |
|  |  |  | Blattallidae | *Blattella germanica* |  |  |  |
|  |  |  |  | *B. latistriga* |  |  |  |
|  |  |  |  | *Opisthoplata orientalis* |  |  |  |
|  |  |  | Eupolyphaga | *Eupolyphaga sinensis* |  |  |  |
|  |  |  |  | *E. thibetana* |  |  |  |
|  |  |  |  | *E. yunnaensis* |  |  |  |
|  |  |  |  | *E. everestians* |  |  |  |
|  |  |  |  | *Polyphaga plancyi* |  |  |  |
|  |  |  | Termitidae | *Odontotermes formosanus* | See the article | | endosymbionts |
|  |  |  |  | *O. hainanensis* |  |  |  |
|  |  |  |  | *O. foveafrons* |  |  |  |
|  |  |  |  | *O. annulicornis* |  |  |  |
|  |  |  |  | *Macrotermes annandalei* |  |  |  |
|  |  |  |  | *M. barneyi* |  |  |  |
|  |  |  |  | *M. yunanensis* |  |  |  |
|  |  |  |  | *M. guangxiensis* |  |  |  |
|  |  |  |  | *M. longimentis* |  |  |  |
|  |  |  |  | *M. latinotus* |  |  |  |
|  |  |  |  | *M. trimorphus* |  |  |  |
|  |  |  |  | *M. choui* |  |  |  |
|  |  |  |  | *M. longiceps* |  |  |  |
|  |  |  |  | *M. menlongensis* |  |  |  |
|  |  |  |  | *M. jinghongensis* |  |  |  |
|  |  |  |  | *Globitermes sulphureus* |  |  |  |
|  |  |  |  | *Hypotermes sumatrensis* |  |  |  |
|  |  |  | Rhinotermitidae | *Coptotermes formosanus* |  |  |  |
|  |  |  |  | *C. monsetosus menglunensis* |  |  |  |
|  |  |  |  | *Reticulitermes flaviceps* |  |  |  |
|  |  |  |  | *R. grandis* |  |  |  |
|  |  |  |  | *Parrhinotermes khasii* |  |  |  |
|  |  | Mantodea | Mantidae | *Paratenodera sinensis* | See the article | | endogenous |
|  |  |  |  | *P. augustipennis* |  |  |  |
|  |  |  |  | *Mantis religiosa* |  |  |  |
|  |  |  |  | *Statilia maculata* |  |  |  |
|  |  |  |  | *S. nemoralis* |  |  |  |
|  |  |  |  | *Tenodera sinensis* |  |  |  |
|  |  |  |  | *T. aridifolia* |  |  |  |
|  |  |  |  | *T. angustipennis* |  |  |  |
|  |  |  |  | *Hierodula patellifera* |  |  |  |
|  |  |  |  | *H. membranacea* |  |  |  |
|  |  | Orthoptera | Acrididae | *Oxya chinensis* | hirsutellic acid A (IC_50_ = 8 µM, *Plasmodium falciparum*) | | (Isaka et al., 2006)  endosymbionts |
|  |  |  |  | *Acrida lata* |  |  |  |
|  |  |  |  | *A. cinerea* |  |  |  |
|  |  |  |  | *Locusta migratoria manilensis* |  |  |  |
|  |  |  |  | *Ceracris kiangsu* |  |  |  |
|  |  |  |  | *Patanga japonca* |  |  |  |
|  |  |  | Gryllidae | *Gryllus chinensis* |  |  |  |
|  |  |  |  | *G. testaceus* |  |  |  |
|  |  |  |  | *G. mitratus* |  |  |  |
|  |  |  |  | *Loxoblemmus doenitzi* |  |  |  |
|  |  |  |  | *Velarifictorus aspersus* |  |  |  |
|  |  |  |  | *V. micado* |  |  |  |
|  |  |  | Tettigoniidae | *Mecopoda elongata* |  |  |  |
|  |  |  |  | *Gampsocleis buergeri* |  |  |  |
|  |  |  | Gryllotalpidae | *Gryllotalpa africana* | See the article | | endogenous |
|  |  |  |  | *G. orientalis* |  |  |  |
|  |  |  |  | *G. uncspina* |  |  |  |
|  |  | Hemiptera | Cicadidae | *Cryptotympana pustulata* | See the article | | endogenous |
|  |  |  |  | *Cryptotympana atrata* |  |  |  |
|  |  |  |  | *Cryptotympana mandarina* |  |  |  |
|  |  |  |  | *Cryptotympana tustulata* |  |  |  |
|  |  |  |  | *Oncotympana maculaticollis* |  |  |  |
|  |  |  |  | *Hyalessa ronsnana* |  |  |  |
|  |  |  |  | *Oncotympana sp.* |  |  |  |
|  |  |  |  | *Cicadatra shaluensis* |  |  |  |
|  |  |  |  | *Cicada flammata* |  |  |  |
|  |  |  |  | *Mogannia conica* |  |  |  |
|  |  |  |  | *Platypleura kaempferi* |  |  |  |
|  |  |  |  | *Huechys sanguinea* |  |  |  |
|  |  |  |  | *H. philamata* |  |  |  |
|  |  |  |  | *H. thoracica* |  |  |  |
|  |  |  | Pemphigidae | *Schlechtendalia chinensis* | See the article | | aphid parasites |
|  |  |  |  | *S. peita* |  |  |  |
|  |  |  |  | *Nurudea sinica* |  |  |  |
|  |  |  |  | *N. shiraii* |  |  |  |
|  |  |  |  | *N. rosea* |  |  |  |
|  |  |  |  | *Kaburagia ensigallis* |  |  |  |
|  |  |  |  | *K. ovogallis* |  |  |  |
|  |  |  |  | *K. rushicola* |  |  |  |
|  |  |  |  | *K. ovatirhusicola* |  |  |  |
|  |  |  |  | *Meitanaphis elongallis* |  |  |  |
|  |  |  |  | *M. flavogallis* |  |  |  |
|  |  |  |  | *M. microgallis* |  |  |  |
|  |  |  |  | *Floraphis meitanaensis* |  |  |  |
|  |  |  |  | *F. choui* |  |  |  |
|  |  |  | Coccidae | *Ericerus pela* | See the article | | endogenous |
|  |  |  | Lacciferidae | *Kerria yunnanensis* |  |  |  |
|  |  |  |  | *K. lacca* |  |  |  |
|  |  |  |  | *K. chinensis* |  |  |  |
|  |  |  |  | *K. nepalensis* |  |  |  |
|  |  |  |  | *K. pusana* |  |  |  |
|  |  |  |  | *K. ruralis* |  |  |  |
|  |  |  |  | *K. sindica* |  |  |  |
|  |  |  |  | *Metatachardin myrica* |  |  |  |
|  |  |  | Fulgoridae | *Lycorma delicatula* | Unknown | | |
|  |  |  | Pentatomidae | *Aspongopus chinensis* | See the article | | endogenous |
|  |  |  |  | *Tessaratoma papillosa* |  |  |  |
|  |  |  |  | *T. quadrata* |  |  |  |
|  |  |  |  | *Cyclopelta parva* |  |  |  |
|  |  |  | Gerridae | *Aquarium paludum* | Unknown | | |
|  |  |  | Belostomatidae | *Lethocerus indicus* | Unknown | | |
|  | **Holometabola**  **Holometabola**  **Holometabola**  **Holometabola**  **Holometabola**  **Holometabola**  **Holometabola**  **Holometabola** | Coleoptera | Carabidae | *Pheropsophus jessoensis* | Unknown | | |
|  |  |  | Dytiscidae | *Cybister tripunctatus* | Unknown | | |
|  |  |  |  | *C. japonicus* |  |  |  |
|  |  |  |  | *C. limbatus* |  |  |  |
|  |  |  | Gyrinidae | *Gyrinus curtus* | Unknown | | |
|  |  |  | Meloidae | *Mylabris phalerata* | See the article | | endogenous |
|  |  |  |  | *M. cichorii* |  |  |  |
|  |  |  |  | *M. schonherri* |  |  |  |
|  |  |  |  | *M. calida pallas* |  |  |  |
|  |  |  |  | *Epicauta gorhami* |  |  |  |
|  |  |  |  | *E. ruficeps* |  |  |  |
|  |  |  |  | *E. aptera* |  |  |  |
|  |  |  |  | *E. caraganae* |  |  |  |
|  |  |  |  | *Hycleus cichorii* |  |  |  |
|  |  |  |  | *Hycleus phaleratus* |  |  |  |
|  |  |  |  | *Meloe coaretatus* |  |  |  |
|  |  |  | Staphylinidae | *Paederus fuscipes* | See the article | | endosymbionts |
|  |  |  |  | *P. tamulus* |  |  |  |
|  |  |  |  | *P. densipennis* |  |  |  |
|  |  |  |  | *P. parallelus* |  |  |  |
|  |  |  |  | *P. tibelenus* |  |  |  |
|  |  |  | Elateridae | *Pleonomus canaliculatus* | Unknown | | |
|  |  |  |  | *Agriotes fuscicollis* |  |  |  |
|  |  |  |  | *Agriotes sericeus* |  |  |  |
|  |  |  |  | *Melanotus caudes* |  |  |  |
|  |  |  |  | *Selatosomus latus* |  |  |  |
|  |  |  | Scarabaeidae | *Catharsius molossus* | N-acetyldopamine dimers  (COX-1: IC_50_=78.85 µM;COX-2: IC_50_=6.43µM) | | (Lu et al., 2015)  endogenous/ endosymbionts |
|  |  |  |  | *Scarabaeus sacar* |  |  |  |
|  |  |  |  | *Copris ochus* |  |  |  |
|  |  |  | Geotrupidae | *Geotrupes laevistriatus* | Unknown | | |
|  |  |  |  | *G. substriatellus* |  |  |  |
|  |  |  | Dynastidae | *Allomyrina dichotoma* | dicotastin Allomyrinanoid A | | (Niu et al., 2016)  endogenous |
|  |  |  |  | *Alissonotum crassum* |  |  |  |
|  |  |  |  | *Eupatorus hardwicki* |  |  |  |
|  |  |  | Lyctidae | *Lyctus brunneus* | Unknown | | |
|  |  |  | Lampyridae | *Luciola chinensis* | Unknown | | |
|  |  |  |  | *L. ficta* |  |  |  |
|  |  |  |  | *L. vitticollis* |  |  |  |
|  |  |  | Melolonthidae | *Holotrichia diomphalia* | 1. holotricin (IC_50_=2.5 µg/mL, E. coli ATCC2592) 2. elaiophylin | | (Lee et al., 1994; Guo et al, 2015)  endogenous |
|  |  |  |  | *H. oblila* |  |  |  |
|  |  |  |  | *H. sauteri* |  |  |  |
|  |  |  |  | *H. parallela* |  |  |  |
|  |  |  | Rutelidae | *Anomala corpulenta* |  |  |  |
|  |  |  | Cetoniidae | *Oxycetonia jucunda* |  |  |  |
|  |  |  |  | *Protaetia brevitarsis* |  |  |  |
|  |  |  | Cerambycidae | *Apriona germari* | Unknown | | |
|  |  |  |  | *Anoplophora chinensis* |  |  |  |
|  |  |  |  | *Anoplophora glabripennis* |  |  |  |
|  |  |  |  | *Batocera horsfieldi* |  |  |  |
|  |  |  | Tenebrionidae | *Martianus dermestoides* | rynchopeterines A–E (IC50=7.67-12.3µg/mL, DPPH ) | | (Xiao et al., 2017)  endogenous |
|  |  |  |  | *Blaps rynchopetera* |  |  |  |
|  |  |  |  | *Tenebrio molitor* |  |  |  |
|  |  |  | Silphidae | *Nicrophorus sp.* | Unknown | | |
|  |  |  | Curculionidae | *Otidognathus davidi* | Unknown | | |
|  |  |  |  | *Cyrtotrachelus longimanus* |  |  |  |
|  |  | Neuroptera | Myrmeleontidae | *Euroleon sinicus* | Unknown | | |
|  |  |  |  | *Myrmeleon micans* |  |  |  |
|  |  |  |  | *Distoleon yunnaus* |  |  |  |
|  |  |  |  | *Palpares sinicus* |  |  |  |
|  |  |  |  | *Epicanthaclisis continentalis* |  |  |  |
|  |  |  |  | *Glenuroides japonicus* |  |  |  |
|  |  | Trichoptera | Phrygancidae | *Phryganea japonica* | Unknown | | |
|  |  | Lepidoptera | Hepialidae | *Hepialus armoricanus* | See the article | | endosymbionts |
|  |  |  |  | *H. kangdinensis* |  |  |  |
|  |  |  |  | *H. baimaensis* |  |  |  |
|  |  |  |  | *H. yushuensis* |  |  |  |
|  |  |  |  | *H. oblifurcus* |  |  |  |
|  |  |  |  | *H. albipictus* |  |  |  |
|  |  |  |  | *H. menyuanicus* |  |  |  |
|  |  |  |  | *H. sichuanus* |  |  |  |
|  |  |  |  | *H. kangdingroides* |  |  |  |
|  |  |  |  | *H. yunlongensis* |  |  |  |
|  |  |  |  | *H. lijiangensis* |  |  |  |
|  |  |  |  | *H. zhangmoensis* |  |  |  |
|  |  |  |  | *H. zhayuensis* |  |  |  |
|  |  |  |  | *H. ganna* |  |  |  |
|  |  |  |  | *H. macilentus* |  |  |  |
|  |  |  |  | *H. renzhiensis* |  |  |  |
|  |  |  |  | *H. altaicola* |  |  |  |
|  |  |  |  | *Palpifer sexnotatus* |  |  |  |
|  |  |  |  | *P. signifer* |  |  |  |
|  |  |  |  | *P. excrescens* |  |  |  |
|  |  |  |  | *P. yunnanensis* |  |  |  |
|  |  |  |  | *P. anhuiensis* |  |  |  |
|  |  |  |  | *P. xizangensis* |  |  |  |
|  |  |  |  | *P. jingdongensis* |  |  |  |
|  |  |  |  | *P. giganodus* |  |  |  |
|  |  |  |  | *P. fujianodus* |  |  |  |
|  |  |  |  | *P. nodus* |  |  |  |
|  |  |  |  | *P. miniatus* |  |  |  |
|  |  |  |  | *P. regius* |  |  |  |
|  |  |  |  | *Hepialiscus nepalensis* |  |  |  |
|  |  |  |  | *Hepialiscus sylvinus* |  |  |  |
|  |  |  |  | *Hepialiscus flavus* |  |  |  |
|  |  |  |  | *Forkalus xizangensis* |  |  |  |
|  |  |  |  | *Bipectilus yunnanensis* |  |  |  |
|  |  |  |  | *Napialus hunanensis* |  |  |  |
|  |  |  |  | *Thitarodes zhangmoensis* |  |  |  |
|  |  |  |  | *Thitarodes xiaojinensis* |  |  |  |
|  |  |  | Eucleidae | *Cnidocampa flavescens* | Unknown | | |
|  |  |  |  | *Thosea sinensis* |  |  |  |
|  |  |  |  | *T. grandis* |  |  |  |
|  |  |  |  | *T. sythoffi* |  |  |  |
|  |  |  |  | *T. loesa* |  |  |  |
|  |  |  | Pyralidae | *Ostrinia furnacalis* | Unknown | | |
|  |  |  |  | *Procera venoosatum* |  |  |  |
|  |  |  |  | *Aglossa dimidiate* |  |  |  |
|  |  |  |  | *Herculia glaucinalis* |  |  |  |
|  |  |  |  | *Hydrillodes morosa* |  |  |  |
|  |  |  |  | *H. repugnalis* |  |  |  |
|  |  |  |  | *Nodaria niphona* |  |  |  |
|  |  |  |  | *Ostrinia nubilalis* |  |  |  |
|  |  |  | Noctuidae | *Hydrillodes morosa* | Unknown | | |
|  |  |  |  | *Nodaria niphona* |  |  |  |
|  |  |  |  | *Agrotis ypsilon* |  |  |  |
|  |  |  | Arctiidae | *Arctia caja* | Unknown | | |
|  |  |  | Brahmaeidae | *Brahmophthalma japonica* | Unknown | | |
|  |  |  | Psychidae | *Cryptothelea formosicola* | Unknown | | |
|  |  |  | Saturniidae | *Philosmia cynthia ricinia* | See the article | | endogenous |
|  |  |  |  | *Antheraea pernyi* |  |  |  |
|  |  |  | Bombycidae | *Bombyx mori* | See the article | | endogenous |
|  |  |  | Geometridae | *Biston robustum* | Unknown | | |
|  |  |  |  | *Biston marginata* |  |  |  |
|  |  |  | Notodontidae | *Phalera bucephala* | Unknown | | |
|  |  |  |  | *P. assimilis* |  |  |  |
|  |  |  |  | *Leucodonata bicoloria* |  |  |  |
|  |  |  |  | *Notodonata dembowskii* |  |  |  |
|  |  |  | Sphingidae | *Smerinthus planus* | Unknown | | |
|  |  |  |  | *Clanis bilineata* |  |  |  |
|  |  |  |  | *C. deucalion* |  |  |  |
|  |  |  | Xyloryctidae | *Linoclostis gonatias* | Unknown | | |
|  |  |  | Pieridae | *Pieris brassicae* | Unknown | | |
|  |  |  |  | *Pieris rapae* |  |  |  |
|  |  |  |  | *Catopsilia crocalc* |  |  |  |
|  |  |  | Papilionidae | *Pailio machaon* | Unknown | | |
|  |  |  |  | *P. xuthus* |  |  |  |
|  |  | Diptera | Tabanidae | *Hermetia illucens* | See the article | endogenous | |
|  |  |  |  | *Tabanus pleskei* |  |  |  |
|  |  |  |  | *T. atratus* |  |  |  |
|  |  |  |  | *T. bivittatus* |  |  |  |
|  |  |  |  | *T. buddha* |  |  |  |
|  |  |  |  | *T. chrysarus* |  |  |  |
|  |  |  |  | *T. mandarinus* |  |  |  |
|  |  |  |  | *T. pleskei* |  |  |  |
|  |  |  |  | *T. trigonus* |  |  |  |
|  |  |  |  | *T. kiangsuensis* |  |  |  |
|  |  |  |  | *Atylotus rusticus* |  |  |  |
|  |  |  |  | *A. bivittateinus* |  |  |  |
|  |  |  | Calliphoridae | *Lucilia sericata* | See the article | endogenous | |
|  |  |  |  | *Chrysomya megacephala* |  |  |  |
|  |  | Hymenoptera | Scoliidae | *Scolia vittifornis* | Venomic protein   1. mastoparan (IC50 = 10 µM, inositol phosphate) 2. phospholipase (PLA1 and PLA2) 3. kinins | (Yokokawa et al., 1989;Nakahata et al., 1990; Mendes and Palma, 2006;Monteiro et al., 2009)  endogenous | |
|  |  |  |  | *Campsomeris annulata* |  |  |  |
|  |  |  | Xylocopidae | *Platynopoda magnifica* |  |  |  |
|  |  |  |  | *Xylocopa sinensis* |  |  |  |
|  |  |  |  | *X. appendiculata* |  |  |  |
|  |  |  | Eumenidae | *Eumenes mediterraneus* |  |  |  |
|  |  |  |  | *E. pomiformis* |  |  |  |
|  |  |  | Polistidae | *Polistes jokahamae* |  |  |  |
|  |  |  |  | *P. chinensis* |  |  |  |
|  |  |  |  | *P. antennalis* |  |  |  |
|  |  |  |  | *P. japonicus* |  |  |  |
|  |  |  |  | *P. olivaceus* |  |  |  |
|  |  |  |  | *P. mandarinus* |  |  |  |
|  |  |  | Polybiidae | *Parapolybia varia varia* |  |  |  |
|  |  |  | Vespidae | *Orancistrocerus drewseni* |  |  |  |
|  |  |  |  | *Vespa nigrithorax* |  |  |  |
|  |  |  |  | *V. ducalis* |  |  |  |
|  |  |  |  | *V. analisparalleia* |  |  |  |
|  |  |  |  | *V. basalis* |  |  |  |
|  |  |  |  | *V. binghami* |  |  |  |
|  |  |  |  | *V. bicolor* |  |  |  |
|  |  |  |  | *V. affinis* |  |  |  |
|  |  |  |  | *V. crabro crabro* |  |  |  |
|  |  |  |  | *V. magnifica* |  |  |  |
|  |  |  |  | *V. mandarinia* |  |  |  |
|  |  |  |  | *V. simillima* |  |  |  |
|  |  |  |  | *V. tropica leefmansi* |  |  |  |
|  |  |  |  | *Protopolybia exigua* |  |  |  |
|  |  |  |  | *Polybia occidentalis* |  |  |  |
|  |  |  | Formicidae | *Polyrhachis lamellidens* | See the article | endogenous | |
|  |  |  |  | *P. vicina* |  |  |  |
|  |  |  |  | *P. dives* |  |  |  |
|  |  |  |  | *P. paracamponota* |  |  |  |
|  |  |  |  | *P. furcata* |  |  |  |
|  |  |  |  | *P. rastellata* |  |  |  |
|  |  |  |  | *P. bihamata* |  |  |  |
|  |  |  |  | *Formica fusca* |  |  |  |
|  |  |  |  | *F. sanguinea* |  |  |  |
|  |  |  |  | *F. rufa* |  |  |  |
|  |  |  |  | *F. approximans* |  |  |  |
|  |  |  |  | *F. yessensis* |  |  |  |
|  |  |  |  | *Tetramorium bicarinatum* |  |  |  |
|  |  |  |  | *Oecophylla smaragdina* |  |  |  |
|  |  |  |  | *Odontoponera transversa* |  |  |  |
|  |  |  |  | *Bothroponera rufipes* |  |  |  |
|  |  |  |  | *Phidologiton offinis* |  |  |  |
|  |  |  |  | *Tetramorium guineense* |  |  |  |
|  |  |  |  | *Camponotus japonicus* |  |  |  |
|  |  |  | Apidae | *Apis mellifera caucasica* | See the article | endogenous | |
|  |  |  |  | *A. florea* |  |  |  |
|  |  |  |  | *A. andreniformis* |  |  |  |
|  |  |  |  | *A. laboriosa* |  |  |  |
|  |  |  |  | *A. cerana* |  |  |  |
|  |  |  |  | *A. dorsata* |  |  |  |
|  |  |  |  | *A. mellifera* |  |  |  |
|  |  |  | Cynipidae | *Cynips gallaetinctoriae* | See the article | wasp parasites | |

Note: The insect species marked with yellow color denotes that this insect has been explored for their active pharmaceutical agents during the past decades.

**References**

1. Guo, Z.K., Liu, S.B., Ma, S., and Wang, R. (2015). Antibacterial Metabolites from the Mycelia of the Cockchafer-Derived Streptomyces sp. BCa1. *Chin J Trop Crop* 36**,** 1307-1311.
2. Isaka, M., Kittakoop, P., Kirtikara, K., Hywel-Jones, N.L., and Thebtaranonth, Y. (2005). Bioactive substances from insect pathogenic fungi. *Acc Chem Res* 38**,** 813-823.
3. Lee, S.Y., Moon, H.J., Kurata, S., Kurama, T., Natori, S., and Lee, B.L. (1994). Purification and molecular cloning of cDNA for an inducible antibacterial protein of larvae of a coleopteran insect, Holotrichia diomphalia. *J Biochem* 115**,** 82-86.
4. Lu, J., Sun, Q., Tu, Z.C., Lv, Q., Shui, P.X., and Cheng, Y.X. (2015). Identification of N-Acetyldopamine Dimers from the Dung Beetle Catharsius molossus and Their COX-1 and COX-2 Inhibitory Activities. *Molecules* 20**,** 15589-15596.
5. Monteiro, M.C., Romao, P.R., and Soares, A.M. (2009). Pharmacological perspectives of wasp venom. *Protein Pept Lett* 16**,** 944-952.
6. Nakahata, N., Abe, M.T., Matsuoka, I., and Nakanishi, H. (1990). Mastoparan inhibits phosphoinositide hydrolysis via pertussis toxin-insensitive [corrected] G-protein in human astrocytoma cells. *FEBS Lett* 260**,** 91-94.
7. Niu, L., Gao, J., Li, H., Liu, J., and Yin, W. (2016). Novel skeleton compound Allomyrinanoid A and two purine alkaloids from the adult of Allomyrina dichotoma L. *Bioorg Med Chem Lett* 26**,** 366-369.
8. Thongtan, J., Saenboonrueng, J., Rachtawee, P., and Isaka, M. (2006). An antimalarial tetrapeptide from the entomopathogenic fungus Hirsutella sp. BCC 1528. *J Nat Prod* 69**,** 713-714.
9. Xiao, H., Yin, T.P., Dong, J.W., Wu, X.M., Luo, Q., Luo, J.R., Cai, L., and Ding, Z.T. (2017). Five New Phenolic Compounds with Antioxidant Activities from the Medicinal Insect Blaps rynchopetera. *Molecules* 22.
